# Inflammatory bowel disease and the associated risk of dry eye and ocular surface injury: a nationwide matched cohort study

**DOI:** 10.1186/s12886-023-03165-z

**Published:** 2023-10-13

**Authors:** Yi-Ting Ko, Yu-Ming Wu, Hsiang-Ling Wu, Shih-Chung Lai, Ying-Xiu Dai, Tzeng-Ji Chen, Yih-Giun Cherng, Ying-Hsuan Tai, Chia-Yu Kao

**Affiliations:** 1 Department of Anesthesiology, Shuang Ho Hospital, Taipei Medical University, New Taipei City, 23561 Taiwan; 2 Department of Anesthesiology, School of Medicine, College of Medicine, Taipei Medical University, Taipei, 11031 Taiwan; 3 Department of Anesthesiology, Taipei Veterans General Hospital 11217, Taipei, Taiwan; 4 School of Medicine, National Yang Ming Chiao Tung University, Taipei, 11221 Taiwan; 5 Department of Ophthalmology, Shuang Ho Hospital, Taipei Medical University, New Taipei City, 23561 Taiwan; 6 Department of Ophthalmology, School of Medicine, College of Medicine, Taipei Medical University, Taipei, 11031 Taiwan; 7 Department of Dermatology, Taipei Veterans General Hospital, Taipei, 11217 Taiwan; 8 Department of Family Medicine, Taipei Veterans General Hospital, Taipei, 11217 Taiwan; 9 Department of Family Medicine, Taipei Veterans General Hospital, Hsinchu Branch, Hsinchu, 31064 Taiwan

**Keywords:** Crohn's disease, Keratoconjunctivitis sicca, Peripheral ulcerative keratitis, Ulcerative colitis

## Abstract

**Background:**

Inflammatory bowel disease (IBD) is associated with lacrimal gland dysfunction and ocular inflammation. The objective of this research was to elucidate the temporal relationships between IBD, dry eye disease (DED), and corneal surface damage.

**Methods:**

In a matched nationwide cohort study, we evaluated the risk of DED and corneal surface damage associated with IBD. Multivariable Cox proportional hazards regression analyses were implemented to estimate the risk of ocular complications.

**Results:**

A total of 54,293 matched pairs were included for analyses. The median follow-up time was 8.3 years (interquartile range: 5.5 – 10.5). The period incidence of DED was 8.18 and 5.42 per 1000 person-years in the IBD and non-IBD groups, respectively. After adjusting for confounders, statistically significant associations were found between IBD and DED [adjusted hazard ratio (aHR): 1.43, 95% confidence interval (CI): 1.35 – 1.51, *p* < 0.0001], Sjögren’s syndrome-related (aHR: 1.67, 95% CI:1.46 – 1.90, *p* < 0.0001) and non-Sjögren’s syndrome-related subtypes (aHR: 1.38, 95% CI: 1.30 – 1.46, *p* < 0.0001). Furthermore, increased risks of corneal surface damage (aHR: 1.13, 95% CI: 1.03 – 1.24, *p* = 0.0094) among the patients with IBD were observed when compared with the controls. Other independent factors associated with corneal surface damage were age (aHR: 1.003), sex (male vs. female, aHR: 0.85), and monthly insurance premium (501–800 vs. 0–500 U.S. dollars, aHR: 1.45; ≥ 801 vs. 0–500 U.S. dollars, aHR: 1.32).

**Conclusions:**

Our results suggested that IBD was an independent risk factor for DED and ocular surface damage. Clinical strategies are needed to prevent visual impairment or losses in these susceptible patients.

**Supplementary Information:**

The online version contains supplementary material available at 10.1186/s12886-023-03165-z.

## Background

Dry eye disease (DED) is a highly prevalent disorder and characterized by increased osmolarity of the tear film and inflammation of the ocular surface [1]. Epidemiological studies demonstrated that the prevalence of DED ranged from 5 to 50% worldwide [2]. In Taiwan, the crude incidence rate of DED was reported to be 4.26 per 1000 population in 2015 [3]. In the elderly, the prevalence rate was as high as 33.7% in a Taiwan’s community questionnaire survey [4]. DED can be classified into aqueous-deficient and evaporative subtypes by the Dry Eye Workshop of Tear Film and Ocular Surface (TFOS) Society [5]. The aqueous tear-deficient DED can be further divided into Sjögren's syndrome (SS)-related and non-SS-related subtypes [5, 6]. The evaporative DED presents normal lacrimal secretory functions but excessive water loss from the exposed ocular surface [5, 7]. The symptom of DED has a potential adverse impact on patients' physical function and quality of life [8, 9]. The increased treatment utilization and productivity loss exert a heavy economic burden on patients with DED [9].

Inflammatory bowel disease (IBD) is an immune-related chronic gastrointestinal inflammation condition, which includes two major types, Crohn's disease (CD) and ulcerative colitis (UC) [10]. The prevalence rate of IBD ranged from 2.1% to 12.8% globally, and the incidence rate has been rising in recent years [11]. The pathogenesis of IBD remains unclear. Genetic factors, gut microbial, environment and immunological abnormalities are considered as possible causes [12]. IBD primarily involves the gastrointestinal wall, with continuous lesions of the mucosal and submucosal layer in UC and skipped lesions of the whole layer in CD [13]. In addition to intestinal wall damages and manifestations, IBD may also have a deleterious effect on extraintestinal systems, such as the eye [14]. Previous studies have revealed that nearly 2% to 7% patients with IBD had ocular morbidities with episcleritis, scleritis and uveitis in majority [14]. Czompa et al*. *reported that patients with IBD had higher rates of dry eye and thin cornea with reduced tear quantity compared with non-IBD controls [15]. However, the relationship between IBD and DED remains unclear due to multiple methodological drawbacks of preceding studies, including small patient sample (< 1000 IBD subjects) [15, 16], single-institution settings [15–17], and inadequate adjustment for confounding [15–17]. Importantly, the long-term risk and epidemiological statistics of ocular surface damage in IBD have not been estimated in previous studies.

In a nationwide matched cohort study, we aimed to examine the association between IBD, DED, and corneal surface damage using the administrative data from Taiwan's National Health Insurance (NHI) research database. Based on existing evidence [14–17], we hypothesized that IBD was significantly associated with more DED and corneal surface damage compared with non-IBD people.

## Methods

### Data source

This study was evaluated and approved by Taipei Medical University – Joint Institutional Review Board (TMU-JIRB-N202210011). This study was conducted in accordance with the Helsinki Declaration and the STROBE study guidelines [18]. Written informed consent was waived due to the use of decoded and scrambled beneficiary identifications. The Taiwan government launched a single-payer NHI program in March 1995. Currently, there are more than 23 million Taiwanese residents covered by this program, representing approximately 99.6% of Taiwan’s entire population. A comprehensive description of the NHI research database has been given in previous articles and government’s official websites [19–23].

### Subject eligibility criteria

The participants were considered as having developed an IBD only if the diagnosis was established by board-certified physicians, and the condition occurred at ≥ 2 outpatient visits between January 1, 2002 and June 30, 2013. The diagnostic codes used for this study were based on the International Classification of Diseases, 9th Revision, Clinical Modification (ICD-9-CM) (Supplementary Table S1). The index date for the IBD group was the date when IBD was diagnosed for the first time, whereas the index date for the non-IBD group was the IBD-diagnosed date of the matched IBD subject. Participants with a previous diagnosis of dry eye or corneal diseases were excluded from the analysis, including interstitial and deep keratitis, corneal neovascularization, ocular adnexal burns, open wound of eyeball, corneal ulcers, recurrent corneal erosion, and corneal opacity. Subjects who had used eye lubricants before the index date or died during the study period were also excluded.

### Ocular outcomes

The primary outcome assessed was DED, which was defined as the diagnosis established at least twice in conjunction with prescriptions of cyclosporine ophthalmic emulsion (Restasis®) treatment by board-certified ophthalmologists (Supplementary Table S1). In the NHI regulations, ophthalmic cyclosporine can be reimbursed when the Schirmer test score was < 5 mm in 5 min [11]. DED was further classified into Sjögren’s syndrome (SS)-related or non-SS-related subtypes. The secondary outcomes assessed were serious types of ocular surface damages (corneal ulcers, recurrent corneal erosion, and corneal opacity), which were defined as the diagnosis made twice in the ophthalmology care service. Survival times were the corresponding censored observations in subjects without the ocular outcomes. Patient’s status was followed up until December 31, 2013.

### Patient and clinical characteristics

Insurance premium was categorized into 0 to 500, 501 to 800, and ≥ 801 U.S. dollars per month. The ICD-9-CM codes of medical diagnoses within 2 years before the index date were used to ascertain coexisting diseases potentially related to corneal diseases (Supplementary Table S1) [24]. The Charlson comorbidity index score was assessed for clinical prognosis and comorbidity adjustment [25]. The prescription of systemic steroids within 6 months after the index date was also analyzed. The numbers of hospital admissions and emergency room visits within 2 years before the index date were calculated to evaluate the level of healthcare resource use.

### Statistical analysis

Each IBD subject was matched to a non-IBD subject using the greedy matching methodology with a caliper width of 0.2 SDs of the log odds of the calculated propensity score and without replacement to adjust for the distribution of age, sex, and monthly insurance premium between subjects with and without IBD [26]. An absolute standardized mean difference (ASMD) was used to evaluate the baseline patient characteristics between the matched pairs [27]. Imbalance was defined as an ASMD value higher than 0.1. To clarify the independent relationship between IBD, DED, and corneal surface damage, multivariable Cox regression models were utilized to estimate the adjusted hazard ratio (aHR) for the ocular outcomes. The variables controlled in the multivariable model were age, sex, insurance premium, collected coexisting diseases, Charlson comorbidity index score, use of systemic corticosteroids, number of hospitalizations, and number of emergency room visits. In addition, the potential differences in cumulative incidences of DED and corneal surface damage between the two groups were evaluated using the Kaplan–Meier method and log rank tests. A two-sided significance level of 0.05 was used to define a statistically significant difference. All the statistical analyses were implemented using SAS V.9.4 (SAS Institute Inc., Cary, NC, USA).

## Results

Altogether, a total of 54,293 matched pairs with 841,752 person-years of follow-up were included in this cohort study (Supplementary Figure S1). The median follow-up time of the entire cohort was 8.3 years (interquartile range: 5.5 – 10.5). The distributions of age, sex, and monthly insurance premium were well matched without between-group differences (Table 1). Compared with non-IBD subjects, IBD patients had more comorbidities, uses of systemic steroids, and greater emergence room visits.

**Table 1:** Baseline characteristics of subjects with and without inflammatory bowel disease

	**IBD** ***n* = 54,293**		**Non-IBD** ***n* = 54,293**		**ASMD**
**Age (years), mean (SD)**	31.5	21.5	31.5	21.5	< .0001
**Sex, male, *n* (%)**	26,654	49.1	26,654	49.1	< .0001
**Monthly insurance premium (U.S. dollars), *n* (%)**					< .0001
0–500	26,745	49.3	26,745	49.3	
501–800	14,745	27.2	14,745	27.2	
≥ 801	12,803	23.6	12,803	23.6	
**Coexisting diseases, *n* (%)**					
Hypertension	6148	11.3	4643	8.6	0.1718
Diabetes mellitus	2726	5.0	2057	3.8	0.1624
Coronary artery disease	2330	4.3	1515	2.8	0.2459
Chronic obstructive pulmonary disease	2398	4.4	1471	2.7	0.2792
Chronic liver disease	3513	6.5	2482	4.6	0.2026
Chronic kidney disease	239	0.4	218	0.4	0.0509
Cerebrovascular disease	1118	2.1	949	1.8	0.0921
Major depressive disorder	436	0.8	251	0.5	0.3063
Anxiety disorder	4498	8.3	2567	4.7	0.3302
Thyroid disease	680	1.3	368	0.7	0.3417
Sleeping disorder	4679	8.6	2625	4.8	0.3410
Cancer	1026	1.9	730	1.3	0.1907
**Charlson comorbidity index score**					0.0348
0	49,761	91.7	50,450	92.9	
1	3492	6.4	2847	5.2	
2	850	1.6	784	1.4	
≥ 3	190	0.4	212	0.4	
**Use of systemic corticosteroids, *n* (%)**	9344	17.2	6949	12.8	0.1919
**Number of hospitalizations, *n* (%)**					0.0864
0	47,354	87.2	48,940	90.1	
1	5019	9.2	3986	7.3	
2	1233	2.3	821	1.5	
≥ 3	687	1.3	546	1.0	
**Number of emergency room visits, *n* (%)**					0.1569
0	39,119	72.1	42,680	78.6	
1	9560	17.6	7810	14.4	
2	3013	5.6	2197	4.1	
≥ 3	2601	4.8	1606	3.0	

*Abbreviation*: *ASMD* absolute standardized mean difference, *IBD* inflammatory bowel disease, *SD* standard deviation

**Fig. 1:**
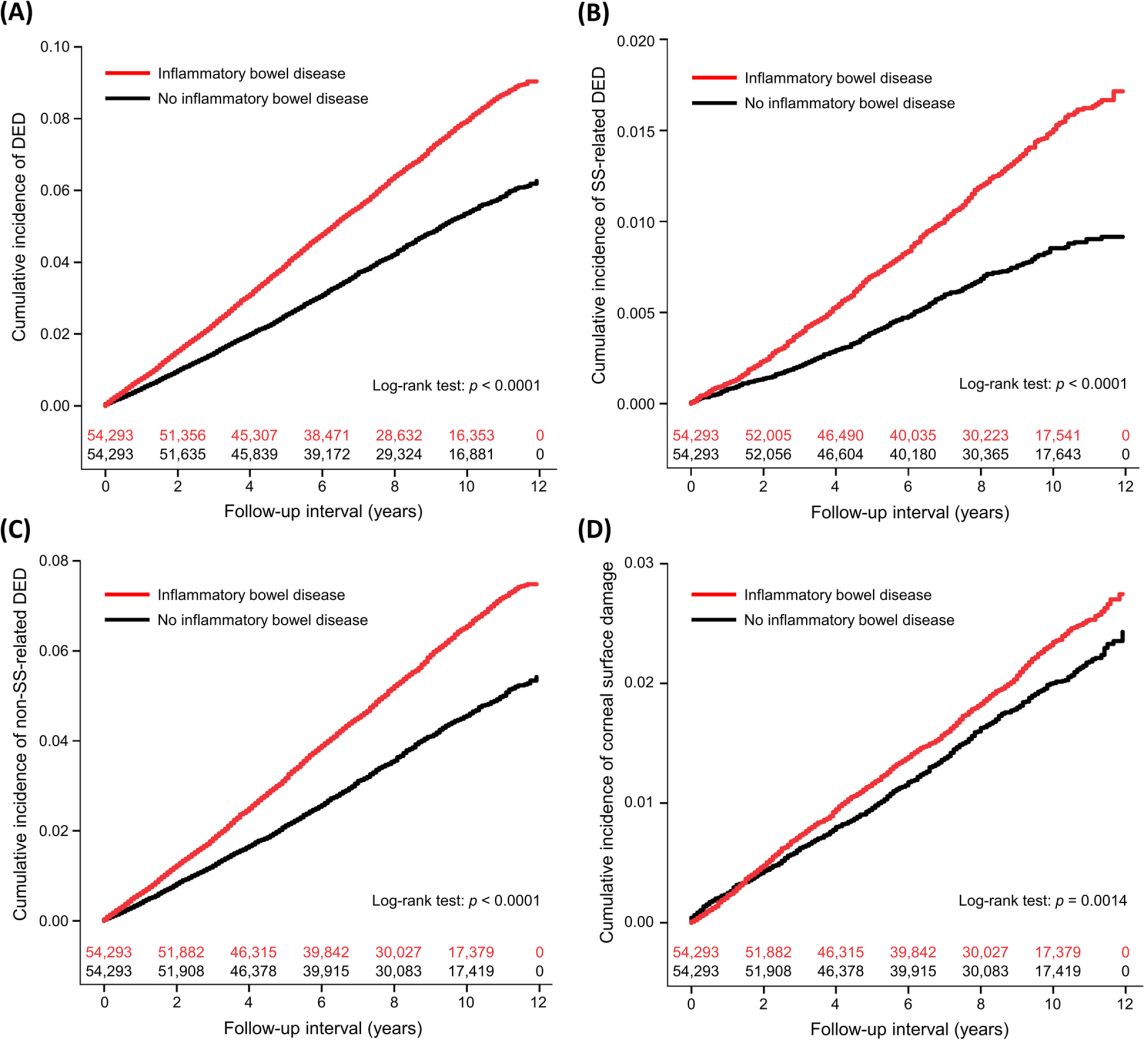
Cumulative risk of dry eye disease (DED) (**A**), Sjögren's syndrome (SS)-related DED (**B**), non-SS-related DED (**C**), and corneal surface damage (**D**) between patients with and without inflammatory bowel disease with number of subjects at risk

In the study period, 3421 patients with IBD developed DED, with a period incidence rate of 8.18 cases per 1000 person-years, whereas 2295 non-IBD controls were diagnosed with DED, with an overall incidence rate of 5.42 cases per 1000 person-years. After adjusting for covariates, patients with IBD had an aHR of 1.43 [95% confidence interval (CI): 1.35 – 1.51, *p* < 0.0001] for DED compared with non-IBD controls (Table 2; Fig. 1A), SS-associated (aHR: 1.67, 95% CI:1.46 – 1.90, *p* < 0.0001; Fig. 1B) and non-SS-associated subtypes (aHR: 1.38, 95% CI: 1.30 – 1.46, *p* < 0.0001; Fig. 1C). The median interval between index date and DED diagnosis was median 4.3 (interquartile range: 2.2 – 6.8) years in the IBD subjects and 4.6 (2.2 – 6.9) years in the non-IBD controls (*p* = 0.0871). Other variables associated with DED were shown in Table 3. Stratified analyses demonstrated that the higher DED risk associated with IBD was significant, independently of different age groups, sex, use of systemic corticosteroids or not, and different comorbidity levels (Table 4).

**Table 2:** Risk of dry eye and ocular surface damage for subjects with and without inflammatory bowel disease

	**IBD** ***n* = 54,293**		**Non-IBD** ***n* = 54,293**		**Outcome risk**		
**Study outcome**	**Incident case**	**Incidence per 1000 person-years**	**Incident case**	**Incidence per 1000 person-years**	**IRR**	**aHR (95% CI)**	***p***
**Dry eye disease**	3421	8.18	2295	5.42	1.51	1.43 (1.35 – 1.51)	< .0001
Sjögren’s syndrome-related	634	1.47	354	0.82	1.79	1.67 (1.46 – 1.90)	< .0001
Non-Sjögren’s syndrome-related	2787	6.67	1941	4.58	1.46	1.38 (1.30 – 1.46)	< .0001
**Corneal surface damage**	1003	2.34	866	2.02	1.16	1.13 (1.03 – 1.24)	0.0094
Corneal ulcer	603	1.41	557	1.30	1.08	1.06 (0.95 – 1.20)	0.2988
Recurrent corneal erosion	212	0.49	137	0.32	1.53	1.52 (1.22 – 1.88)	0.0002
Corneal opacity	188	0.44	173	0.40	1.10	1.04 (0.84 – 1.29)	0.7048

*Abbreviation*: *aHR* adjusted hazard ratio, *CI* confidence interval, *IBD* inflammatory bowel disease, *IRR* incidence rate ratio

**Table 3:** Univariate and multivariable analyses for dry eye disease

	**Univariate**			**Multivariable**		
	**cHR**	**95% CI**	***p***	**aHR**	**95% CI**	***p***
**Inflammatory bowel disease**	1.51	1.43 – 1.59	< .0001	1.43	1.35 – 1.51	< .0001
**Age (years)**	1.036	1.035 – 1.037	< .0001	1.035	1.034 – 1.037	< .0001
**Sex, male vs. female**	0.44	0.42 – 0.47	< .0001	0.49	0.46 – 0.52	< .0001
**Monthly insurance premium (U.S. dollars)**			< .0001			< .0001
501–800 vs. 0–500	1.28	1.21 – 1.37	< .0001	0.97	0.91 – 1.03	0.2823
≥ 801 vs. 0–500	1.32	1.23 – 1.40	< .0001	1.41	1.32 – 1.50	< .0001
**Coexisting diseases**						
Hypertension	2.76	2.59 – 2.95	< .0001	0.91	0.84 – 0.98	0.0176
Diabetes mellitus	2.90	2.66 – 3.16	< .0001	1.25	1.14 – 1.38	< .0001
Ischemic heart disease	3.21	2.94 – 3.52	< .0001	1.16	1.05 – 1.29	0.0033
COPD	2.26	2.05 – 2.50	< .0001	1.11	1.00 – 1.23	0.0625
Chronic liver disease	2.32	2.13 – 2.52	< .0001	1.34	1.23 – 1.46	< .0001
Chronic kidney disease	3.10	2.40 – 4.01	< .0001	1.50	1.15 – 1.95	0.0026
Cerebrovascular disease	2.26	1.97 – 2.60	< .0001	0.78	0.67 – 0.90	0.0009
Thyroid disease	2.64	2.21 – 3.15	< .0001	1.36	1.14 – 1.63	0.0007
Major depressive disorder	2.71	2.18 – 3.36	< .0001	1.30	1.04 – 1.62	0.0201
Anxiety disorder	3.11	2.90 – 3.34	< .0001	1.47	1.36 – 1.60	< .0001
Sleeping disorder	2.89	2.68 – 3.11	< .0001	1.29	1.19 – 1.40	< .0001
Cancer	2.48	2.15 – 2.87	< .0001	1.19	1.02 – 1.38	0.0238
**Charlson comorbidity index score**			< .0001			0.0002
1 vs. 0	1.99	1.83 – 2.16	< .0001	0.85	0.78 – 0.93	0.0003
2 vs. 0	1.93	1.65 – 2.25	< .0001	0.84	0.71 – 0.99	0.0340
≥ 3 vs. 0	1.39	0.96 – 2.01	0.0828	0.66	0.46 – 0.96	0.0295
**Use of systemic corticosteroids**	1.38	1.29 – 1.47	< .0001	1.20	1.13 – 1.28	< .0001
**Number of hospitalizations**			< .0001			< .0001
1 vs. 0	1.23	1.12 – 1.34	< .0001	0.99	0.90 – 1.09	0.8862
2 vs. 0	1.18	0.98 – 1.42	0.0910	0.75	0.62 – 0.91	0.0042
≥ 3 vs. 0	1.09	0.85 – 1.41	0.5013	0.59	0.45 – 0.77	0.0001
**Number of emergency room visits**			0.5482			0.5150
1 vs. 0	0.98	0.91 – 1.06	0.6394	0.96	0.89 – 1.04	0.3348
2 vs. 0	0.95	0.83 – 1.08	0.4028	0.92	0.80 – 1.05	0.2296
≥ 3 vs. 0	1.08	0.94 – 1.24	0.2958	0.95	0.82 – 1.10	0.4879

*Abbreviation*: *aHR* adjusted hazard ratio, *COPD* chronic obstruction pulmonary disease, *cHR* crude hazard ratio

**Table 4:** Stratified analyses of dry eye disease for subjects with and without inflammatory bowel disease

	**IBD** ***n* = 54,293**		**Non-IBD** ***n* = 54,293**		**Outcome risk**		
**Subgroup**	**Incident case**	**Incidence per 1000 person-years**	**Incident case**	**Incidence per 1000 person-years**	**IRR**	**aHR (95% CI)**	***p***
**All patients**	3421	8.18	2295	5.42	1.51	1.43 (1.35 – 1.51)	< .0001
**Age group, years**							
0–19	259	1.67	186	1.20	1.40	1.36 (1.12 – 1.64)	0.0015
20–39	813	6.97	524	4.44	1.57	1.49 (1.33 – 1.67)	< .0001
40–59	1419	1.17	926	0.74	1.57	1.46 (1.34 – 1.58)	< .0001
≥ 60	930	1.72	659	1.19	1.45	1.32 (1.19 – 1.46)	< .0001
**Sex**							
Male	1034	5.02	679	3.27	1.54	1.43 (1.30 – 1.58)	< .0001
Female	2387	11.26	1616	7.48	1.51	1.43 (1.34 – 1.52)	< .0001
**Charlson comorbidity index**							
0	2881	7.55	2001	5.10	1.48	1.42 (1.34 – 1.51)	< .0001
1	431	15.40	215	9.17	1.68	1.51 (1.28 – 1.78)	< .0001
2	90	13.25	70	11.01	1.20	1.09 (0.78 – 1.50)	0.6223
≥ 3	19	13.13	9	5.16	2.54	2.35 (0.99 – 5.56)	0.0519
**Use of systemic corticosteroids**							
Yes	756	10.21	388	7.02	1.45	1.31 (1.16 – 1.49)	< .0001
No	2665	7.75	1907	5.18	1.50	1.45 (1.37 – 1.54)	< .0001

*Abbreviation*: *aHR* adjusted hazard ratio, *CI* confidence interval, *IBD* inflammatory bowel disease, *IRR* incidence rate ratio

A total of 1003 patients with IBD were diagnosed with corneal surface damage, with an overall incidence rate of 2.34 cases per 1000 person-years, whereas 866 non-IBD controls developed corneal surface damage, with an incidence rate of 2.02 cases per 1000 person-years (Table 2). The multivariable models showed that IBD was significantly associated with increased corneal surface damage (aHR: 1.13, 95% CI: 1.03 – 1.24, *p* = 0.0094; Table 5 and Fig. 1D), especially for recurrent corneal erosion (aHR: 1.52, 95% CI: 1.22 – 1.88, *p* = 0.0002). The median time to corneal surface damage was 4.2 years (interquartile range: 2.0 – 7.0) in the IBD patients and 4.3 years (interquartile range: 1.8 – 7.0) in the non-IBD subjects (*p* = 0.7855). In addition, age (aHR: 1.003), sex (male vs. female, aHR: 0.85), and monthly insurance premium (501–800 vs. 0–500 U.S. dollars, aHR: 1.45; ≥ 801 vs. 0–500 U.S. dollars, aHR: 1.32) were independent factors for corneal surface damage.

**Table 5:** Univariate and multivariable analyses for corneal surface damage

	**Univariate**			**Multivariable**		
	**cHR**	**95% CI**	***p***	**aHR**	**95% CI**	***p***
**Inflammatory bowel disease**	1.16	1.06 – 1.27	0.0014	1.13	1.03 – 1.24	0.0094
**Age (years)**	1.007	1.005 – 1.009	< .0001	1.003	1.001 – 1.006	0.0112
**Sex, male vs. female**	0.83	0.75 – 0.91	< .0001	0.85	0.77 – 0.93	0.0005
**Monthly insurance premium (U.S. dollars)**			< .0001			< .0001
501–800 vs. 0–500	1.54	1.39 – 1.71	< .0001	1.45	1.30 – 1.62	< .0001
≥ 801 vs. 0–500	1.33	1.19 – 1.49	< .0001	1.32	1.18 – 1.49	< .0001
**Coexisting diseases**						
Hypertension	1.40	1.22 – 1.61	< .0001	1.12	0.94 – 1.33	0.2029
Diabetes mellitus	1.49	1.23 – 1.81	< .0001	1.20	0.96 – 1.49	0.1062
Ischemic heart disease	1.57	1.27 – 1.93	< .0001	1.22	0.96 – 1.54	0.1021
COPD	1.22	0.97 – 1.53	0.0861	1.02	0.80 – 1.29	0.8952
Chronic liver disease	1.22	1.01 – 1.47	0.0363	1.00	0.83 – 1.22	0.9699
Chronic kidney disease	1.96	1.14 – 3.38	0.0156	1.60	0.91 – 2.80	0.1032
Cerebrovascular disease	1.06	0.76 – 1.49	0.7222	0.78	0.55 – 1.12	0.1731
Thyroid disease	1.64	1.12 – 2.40	0.0104	1.35	0.92 – 1.97	0.1298
Major depressive disorder	1.40	0.84 – 2.32	0.1950	1.14	0.68 – 1.92	0.6209
Anxiety disorder	1.41	1.19 – 1.66	< .0001	1.16	0.97 – 1.39	0.1075
Sleeping disorder	1.16	0.97 – 1.39	0.1116	0.90	0.74 – 1.09	0.2692
Cancer	1.45	1.05 – 2.00	0.0239	1.24	0.89 – 1.74	0.1989
**Charlson Comorbidity Index score**			0.1209			0.8436
1 vs. 0	1.23	1.04 – 1.46	0.0174	1.02	0.84 – 1.23	0.8809
2 vs. 0	1.09	0.77 – 1.55	0.6222	0.87	0.61 – 1.25	0.4446
≥ 3 vs. 0	1.00	0.48 – 2.11	0.9923	0.85	0.40 – 1.78	0.6575
**Use of systemic corticosteroids**	1.15	1.02 – 1.30	0.0213	1.12	0.99 – 1.26	0.0645
**Number of hospitalizations**			0.3234			0.3827
1 vs. 0	0.99	0.84 – 1.18	0.9212	0.93	0.78 – 1.11	0.3948
2 vs. 0	1.33	0.98 – 1.81	0.0684	1.20	0.87 – 1.65	0.2633
≥ 3 vs. 0	0.92	0.57 – 1.49	0.7411	0.80	0.48 – 1.32	0.3814
**Number of emergency room visits**			0.1998			0.1892
1 vs. 0	1.14	1.01 – 1.29	0.0369	1.14	1.00 – 1.29	0.0435
2 vs. 0	1.02	0.82 – 1.28	0.8404	1.02	0.81 – 1.29	0.8491
≥ 3 vs. 0	0.95	0.74 – 1.24	0.7220	0.93	0.70 – 1.22	0.5904

*Abbreviation*: *aHR* adjusted hazard ratio, *COPD* chronic obstruction pulmonary disease, *cHR* crude hazard ratio

## Discussion

In this cohort study, patients with IBD had a significantly higher risk of DED and secondary SS than non-IBD individuals. Additionally, we also observed a significant association between IBD and ocular surface damage, especially for recurrent corneal erosion. Our analyses demonstrated several clinical factors associated with corneal injury, providing an important implication in early identification and intervention of severe ocular morbidities in IBD patients. To the best of our knowledge, this is the first large population-based study to evaluate the association between IBD and corneal diseases. Our findings highlight an urgent need for regular ophthalmology surveillance and timely referral to prevent potential vision-threatening complications among patients with IBD.

Although ocular involvement is not uncommon among patients with IBD, few studies have evaluated the long-term temporal relationship between IBD, dry eye, and corneal damage. Most previous studies restricted their study population to single-hospital settings with small patient samples [15–17]. In a single-center prospective study, Czompa et al*. *reported that 30 CD patients and 36 UC patients had thinner cornea with reduced tear quantity compared with 80 controls, while the corneal power did not decrease significantly [15]. In addition, the corneal volume and anterior chamber volume were lower in the UC group [15]. Another study reported that the dry eye rate assessed using the Schimer’s test and tear breakup time was three times higher in the IBD group with increased Ocular Surface Disease Index scores compared with the controls [16]. Furthermore, the ocular surface had squamous metaplasia and absence of goblet cells in patients with IBD [16]. In a retrospective study, Cuny et al*. *reported that the prevalence of ocular morbidity was 2.6% in IBD, and DED affected 1% of IBD patients [17], which was relatively lower than our results (approximately 9% in the 12-year follow-up). The previous studies had small patient samples with cross-sectional design, making it difficult to accurately evaluate the long-term risk estimates and to clearly delineate the temporal relationship of DED and corneal injury related to IBD. In the present study, our sample size was relatively large with a nationwide coverage, and our analyses included a variety of patient and clinical factors, which have not been examined previously.

The pathological pathway of DED in IBD was still unclear. In an animal study, Sekijima et al*. *showed that reduced tear secretion, inflammatory cell infiltration and destruction of lacrimal gland were discovered in a mice model with UC, indicating that the inflammation and functional decline of lacrimal gland are potentially responsible for dry eye in UC [28]. In humans, Dogan et al. reported that both tear production and tear-film quality were lower among IBD patients; goblet cell losses and squamous metaplasia on the ocular surface were evident [16]. These pathological findings might be the results of ocular inflammation [16]. Furthermore, T cells, interleukins and interferons are involved in the inflammatory and apoptosis reactions of lacrimal gland, cornea and conjunctiva, contributing to the development and progress of DED [7]. Some researchers hypothesized that the mechanism of the ocular manifestations in IBD includes two main pathways: the expansion of immune responses from the intestine or an independent ocular inflammatory event [14]. Inflammatory damage of intestinal mucosa facilitates the passage of microorganisms and proteins and activates lymphoid tissue responses, antibody production, antigen–antibody complex formation, and induces systemic inflammation [29]. Microbial pathogens may also play a pathogenic role via molecular mimicry although few studies have focused on this mechanism [30]. Other studies suggested that genetic factors potentially contribute to the ocular morbidities of IBD. Mallas et al. reported that HLA-B27 type leukocytes were more common among CD patients with extraintestinal manifestations compared with the normal population [31]. However, sparse evidence was available to give a definite explanation for the association between IBD and corneal damage. More studies are warranted to elucidate the biological mechanism and immunological processes of corneal diseases in IBD.

Epidemiological studies estimated that the rate of ocular morbidities in IBD ranged from 2 to 7%, occurring more frequently in CD than UC [14, 32]. Some risk factors were reported, including presence of both colitis and ileocolitis and multi-organ involvement (e.g., arthralgia) [33, 34]. Our results suggested that older age and female sex were also risk factors for corneal surface damage, which have not been reported previously. Our results highlight the importance of regular ophthalmology follow-up for potential corneal surface damage in patients with IBD. Early diagnoses and intervention for ocular complications are pivotal in improving the quality of life and reducing the economic burden in IBD patients.

Our study showed that IBD patients had an increased long-term risk of secondary SS compared with general population. Few studies have investigated the relationship between IBD and SS. There were only case series on SS as an extraintestinal manifestations in IBD [35–37]. The etiology of SS in IBD remains unknown nowadays. Mandl et al. has shown that severe dysbiosis was more common in patients with primary SS and was associated with disease activity [30]. In a small cohort, Palm et al. did not find an association between SS and IBD, which might be due to insufficient statistical power [35]. Our results suggested that clinicians should be aware of the risk of secondary SS in IBD patients. Ophthalmology and rheumatology consultations may be helpful in preventing SS-associated complications. Future efforts should be put on elucidating the pathogenesis and prophylactic strategy for IBD-associated SS.

There were some limitations in our study. First, the activity and severity of IBD (e.g., affected bowel segments and extraintestinal organ involvement) were unknow due to data unavailability in the NHI database. Therefore, we could not analyze the relationship between the activity of IBD and risk of corneal damage [38]. Second, we had no information about patients’ daily activities (e.g., physical capacity and sleep duration), physical examination findings, biochemical and laboratory data, pharmacological treatment details (e.g., immunomodulators and biologics), and clinical presentations (e.g., subjective symptoms of DED) of the included patients. Therefore, the interaction between IBD-related medications, immune system functions, and ocular diseases could not be evaluated in this study [38, 39]. Third, we only evaluated three forms of ocular surface damages (corneal ulcers, recurrent corneal erosion, and corneal opacity), which was based on physiology plausibility, data availability, and existing literature [40]. Further studies are needed to clarify whether IBD patients were susceptible to other types of ocular surface injury. Fourth, the propensity-score matching process only included the parameters of age, sex, and monthly insurance premium between the two groups to minimize sample losses and to ensure sufficient statistical power of the matched dataset. A large sample is necessary in evaluating the long-term risk of SS and corneal surface damage associated with IBD because the incidence rates were relatively low. Finally, the subjects were followed up only until the end of 2013 due to the NHI regulations.

## Conclusions

This large population-based cohort study found that patients with IBD had a significantly higher risk of DED, secondary SS, and corneal damage. Gastroenterologists treating patients with IBD should be aware of these possible sight-threatening complications and refer patients with corneal manifestations to ophthalmologists for evaluation and management. More attention should be paid to patients at high risk of corneal injury, such as older, female patients with both colitis and ileocolitis and multiple organ involvement. Prophylactic and therapeutic strategies should be further developed to promote vision health in these susceptible patients.

### Supplementary Information

**Additional file 1: Supplementary Table S1.** ICD-9-CM codes of exposure factor, coexisting diseases, and ocular outcomes.

**Additional file 2: Supplementary Figure S1.** Flow diagram for patient selection.

## Data Availability

The data generated and analyzed during this study are available from the corresponding author on reasonable request.

## Abbreviations

aHR: Adjusted hazard ratio
ASMD: Absolute standardized mean difference
CD: Crohn's disease
CI: Confidence interval
DED: Dry eye disease
IBD: Inflammatory bowel disease
ICD-9-CM: International Classification of Diseases, 9th Revision, Clinical Modification
NHI: National Health Insurance
SS: Sjögren’s syndrome
TFOS: Tear Film and Ocular Surface
UC: Ulcerative colitis
