# Supplementary material for: Inflammatory bowel disease and the associated risk of dry eye and ocular surface injury: a nationwide matched cohort study
Source: BMC Ophthalmol. 2023 Oct 13;23:415. doi: 10.1186/s12886-023-03165-z (PMC10576268; doi:10.1186/s12886-023-03165-z)
Supplement: Supplementary file 1 — Additional file 1: Supplementary Table S1. ICD-9-CM codes of exposure factor, coexisting diseases, and ocular outcomes. [file 12886_2023_3165_MOESM1_ESM.docx]

**Supplementary Table S1.** ICD-9-CM codes of exposure factor, coexisting diseases, and ocular outcomes

| **Exposure factor** | |
| --- | --- |
| Inflammatory bowel disease | 555.0, 555.1, 555.2, 555.9, 556.0, 556.6, 556.8, 556.9 |
| **Exclusion criteria** | |
| Dry eye disease | 370.33, 372.53, 375.15, 710.2 |
| Interstitial and deep keratitis | 370.5 |
| Corneal neovascularization | 370.6 |
| Ocular adnexal burns | 940 |
| Open wound of eyeball | 871 |
| Corneal ulcer | 370.0 |
| Recurrent corneal erosion | 371.42 |
| Corneal opacity | 371.0 |
| **Coexisting disease** | |
| Hypertension | 401-405 |
| Diabetes mellitus | 250 |
| Ischemic heart disease | 410-414 |
| Chronic obstructive pulmonary disease | 490, 491, 496 |
| Chronic liver disease | 571 |
| Chronic kidney disease | 585 |
| Cerebrovascular disease | 430-438 |
| Thyroid disease | 240-246 |
| Major depressive disorder | 296.2, 296.3 |
| Anxiety disorder | 300 |
| Sleeping disorder | 307.41, 307.42, 327.0, 780.50, 780.52, 292.85 |
| Cancer | 140-208, 230-234 |
| **Ocular outcome** | |
| Dry eye disease | 370.33, 372.53, 375.15, 710.2 |
| Sjögren's syndrome | 710.2 |
| Corneal ulcer | 370.0 |
| Recurrent corneal erosion | 371.42 |
| Corneal opacity | 371.0 |
