# Supplementary figures and images for: Inflammatory bowel disease and the associated risk of dry eye and ocular surface injury: a nationwide matched cohort study
Source: BMC Ophthalmol. 2023 Oct 13;23:415. doi: 10.1186/s12886-023-03165-z (PMC10576268; doi:10.1186/s12886-023-03165-z)

**Supplementary Figure S1.** Flow diagram for patient selection


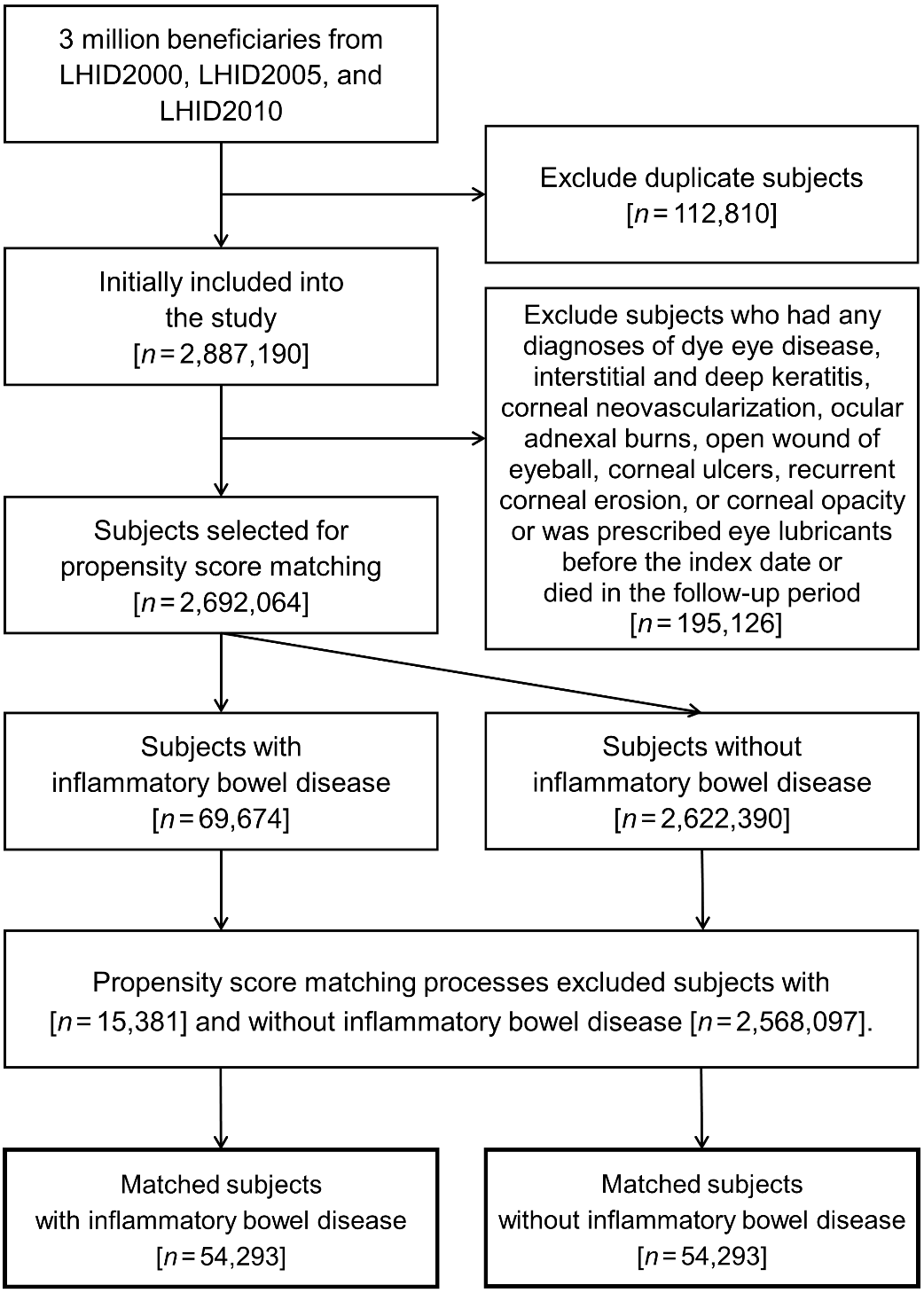

Supplement: Supplementary file 2 — Additional file 2: Supplementary Figure S1. Flow diagram for patient selection. [file 12886_2023_3165_MOESM2_ESM.docx]
